# The future of the past in the present: biodiversity informatics and geological time

**DOI:** 10.3897/zookeys.150.2350

**Published:** 2011-11-28

**Authors:** Edward Baker, Kenneth G. Johnson, Jeremy R. Young

**Affiliations:** 1 Department of Entomology, Natural History Museum, London, United Kingdom; 2 Department of Palaeontology, Natural History Museum, London, United Kingdom; 3 Department of Earth Sciences, University College London, London, United Kingdom

**Keywords:** Palaeontology, Biodiversity Informatics, Scratchpads, web services, GBIF

## Abstract

The biological and palaeontological communities have approached the problem of informatics separately, creating a divide between communities that is both technological and sociological in nature. In this paper we describe one new advance towards solving this problem - expanding the Scratchpads platform to deal with geological time. In creating this system we have attempted to make our work open to existing communities by providing a webservice of geological time data via the GBIF Vocabularies site. We have also ensured that our system can adapt to changes in the definition of geological time intervals and is capable of querying datasets independently of the format of geological age data used.

## Introduction

Over recent years a number of projects have set out to create online communities and resources for the biological community. Similar projects have been developed by the palaeontological community to cover fossil taxa (e.g. http://www.paleodb.org) and to share information associated with geological time (for example http://www.chronos.org/).

Since the overwhelming majority of these resources are focused at workers in either the palaeontological or the neontological communities, a virtual divide is created between communities who work on the same branch of the tree of life. Especially when working with extant taxa that occur in the fossil record or when attempting to compile taxonomic information for both extinct and extant taxa within a particular group (http://corallosphere.org).

In order to address this problem we have taken the Scratchpads platform (http://scratchpads.eu; Smith et al. 2011) and developed additional functionality to allow for the recording of geological age data - a prerequisite for large-scale uptake of the Scratchpads platform by the palaeontological community. It should be noted that our solution deals only with age data and does not attempt to handle stratigraphy, although in well-studied local areas where stratigraphic terms have well-established geochronological meaning (e.g. Blue Lias around Lyme Regis) it is possible to model these names using the system we have developed.

### Handling geological time – the nature of the problem

For a palaeontological database, and indeed most other types of geological data, geological age is an essential data type. For example, one might wish to record the likely age of a specimen or the age range through which a particular species is known to have lived. This sounds like a straightforward databasing problem analogous to recording the age of an historical object or geographical location data; age data or geographical location data can be converted into numerical age or geospatial coordinates on a one-off basis only needing to be revised if the original data is revised.

However, the geological timescale is not a simple known system but a constantly evolving body of knowledge. This can be illustrated by an example – consider the following statement: “The Ichthyosaur was collected from the *Arietites bucklandi* ammonite zone of the Blue Lias, at Lyme Regis (195-196Ma).” The hard data here is that the fossil was collected from the *bucklandi* zone, whilst the geological age given, 195-196Ma, is a modern estimate of the age of that zone. This interpretation has changed in the past and will change in the future as the geological timescale is refined. Changes may occur in this case either because the age of the Lower Jurassic is refined as the whole timescale is re-calibrated in the light of better radiometric data, or because the relative duration of the ammonite zones within it are refined. Indeed, whilst a modern (Gradstein et al. 2004) age estimate for the *bucklandi* zone is 195-196Ma an earlier estimate (Harland et al. 1982) was 205 to 206Ma. So even though the numerical age is needed for communication to non-expert audiences and for database queries, it is essential that only the primary data is recorded in the database and this is then dynamically used to derive the numerical age interpretations as needed, going via a separately maintained look-up table or dictionary of age definitions.

## Community resources

Van Couvering and Ogg (2007) lamented the lack of an online service for geological timescale data. So, as part of the project we have added several sets of geological timescale data to the GBIF Vocabularies site (e.g. http://vocabularies.gbif.org/vocabularies/geo_chronostrat) based on the GTS 2004 (Gradstein et al. 2004).

Each entry in these vocabularies has a name and either a date or date range (defined by a base and top age) as well as additional metadata where appropriate, e.g. FAD/LAD (first/last appearance datums) for nannofossil events. By providing open access to the information, we have provided a platform from which both we and others can start to build web-based timescale tools. Equally importantly since the vocabularies are stored separately from the specimen records they can readily be updated as revised timescales are developed and these revisions will then cascade to all specimen records.

The present implementation of the GBIF Vocabularies site has several issues. The first of these being a requirement for only alphanumeric characters in the name of a term (e.g. a requirement to use LowerJurassic rather Lower Jurassic). Secondly the age metadata can only be exported via the CSV export, and not the XML webservice. GBIF are currently working on improving the Vocabularies site, and we are working closely with them to ensure that the site will be capable of fulfilling our requirements.

## Current scratchpad implementation

Experimental setups were created on two Scratchpads: Nannotax (http://nannotax.org) and the Indo-Pacific Ancient Ecosystems Group (IPAEG: http://ipaeg.org). Both of these examples use a predefined custom content type (GeoTime) to store information about the geological ages that can be referenced by other content types using a nodereference field. The GeoTime content type stores the name of a geological age range or date along with other essential information, including age data and event type (e.g. FAD/LAD), where applicable.

**Figure 1.**
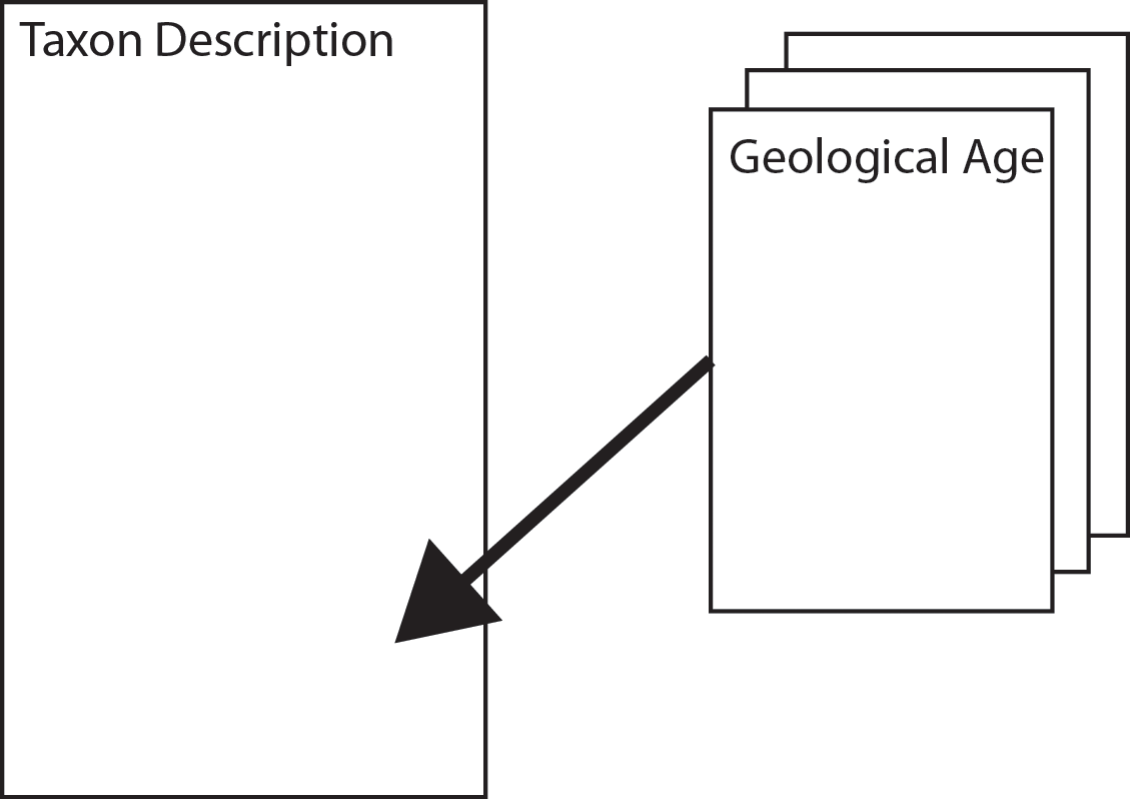
Data model for Nannotax

**Figure 2.**
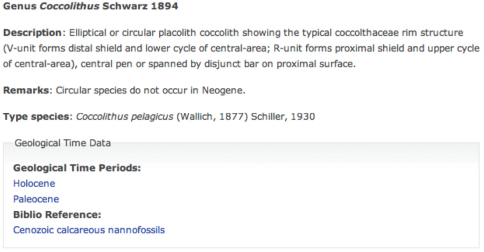
Nannotax Screenshot

**Figure 3.**
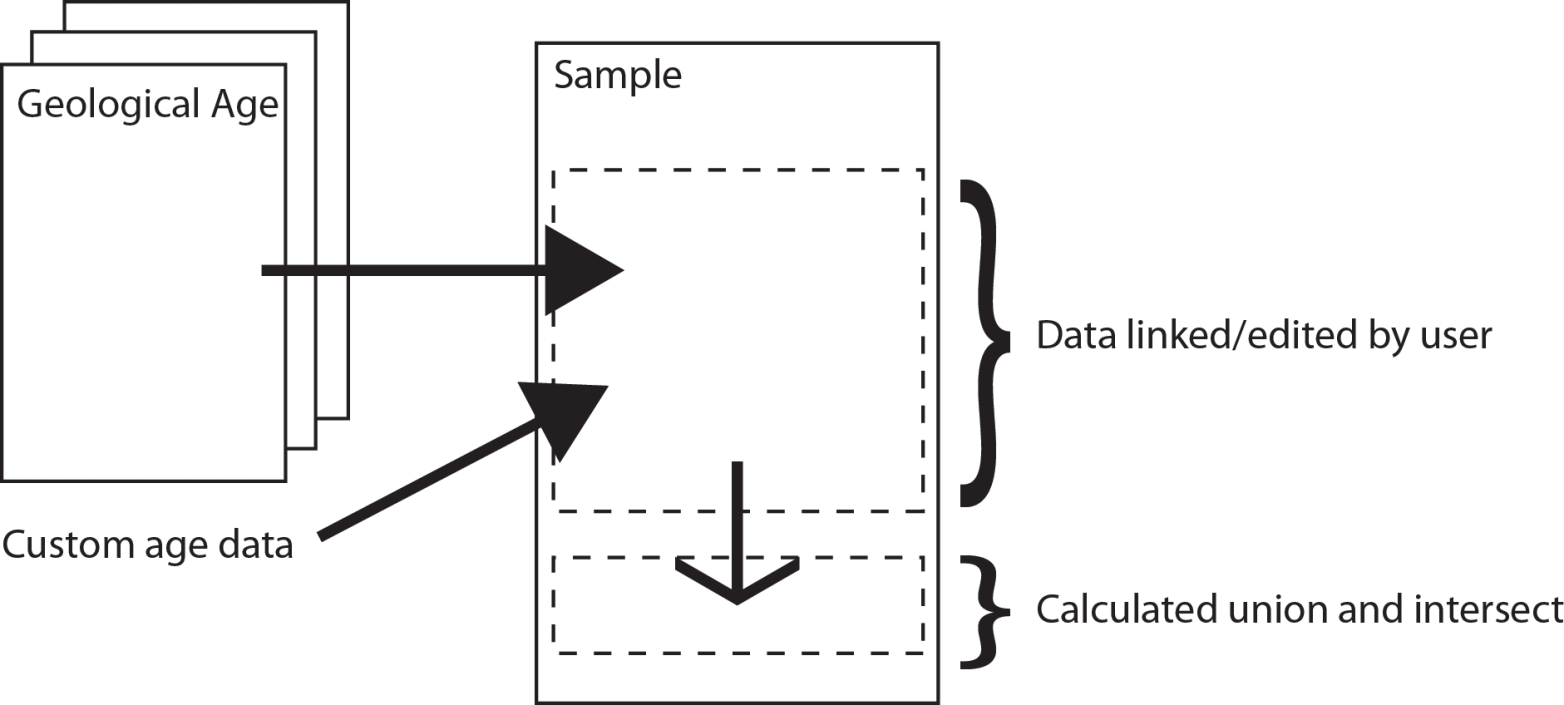
Data model for IPAEG

**Figure 4.**
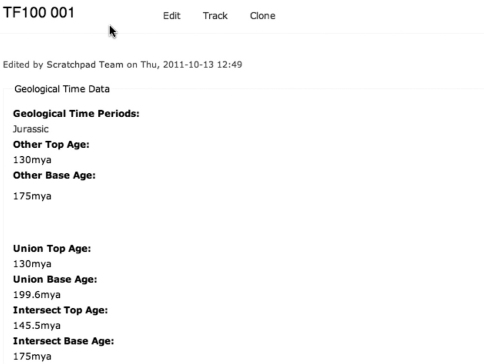
An example Sample record from IPAEG showing user-linked/edited age ranges (above) and calculated union and intersection dates (below).

The Nannotax implementation allows for the first occurrence and last occurrence to be recorded using the data in the form it is available in (e.g. geological stage or magnetochron data). The pair of ages thus defines the total age range of the species and will allow both the age range of the species to be restated and queried in uniform formats (e.g.“which species of taxa X, Y, Z occurred at time n”).

The IPAEG site uses a more complex data model that acts as the foundation point for the Scratchpads 2.0 implementation in development. Like the Nannotax site it is possible to enter any number of predetermined geological ages but, in addition, it is possible to enter a custom age range or custom spot date.

In order to perform calculations with age data it is essential to access the combined range of the data entered by the user. Two useful combinations have been incorporated into the system so far: the union and intersect of the complete data set. Future work may allow specified data to be acknowledged and referenced but excluded from the calculations.

**Figure 5.**
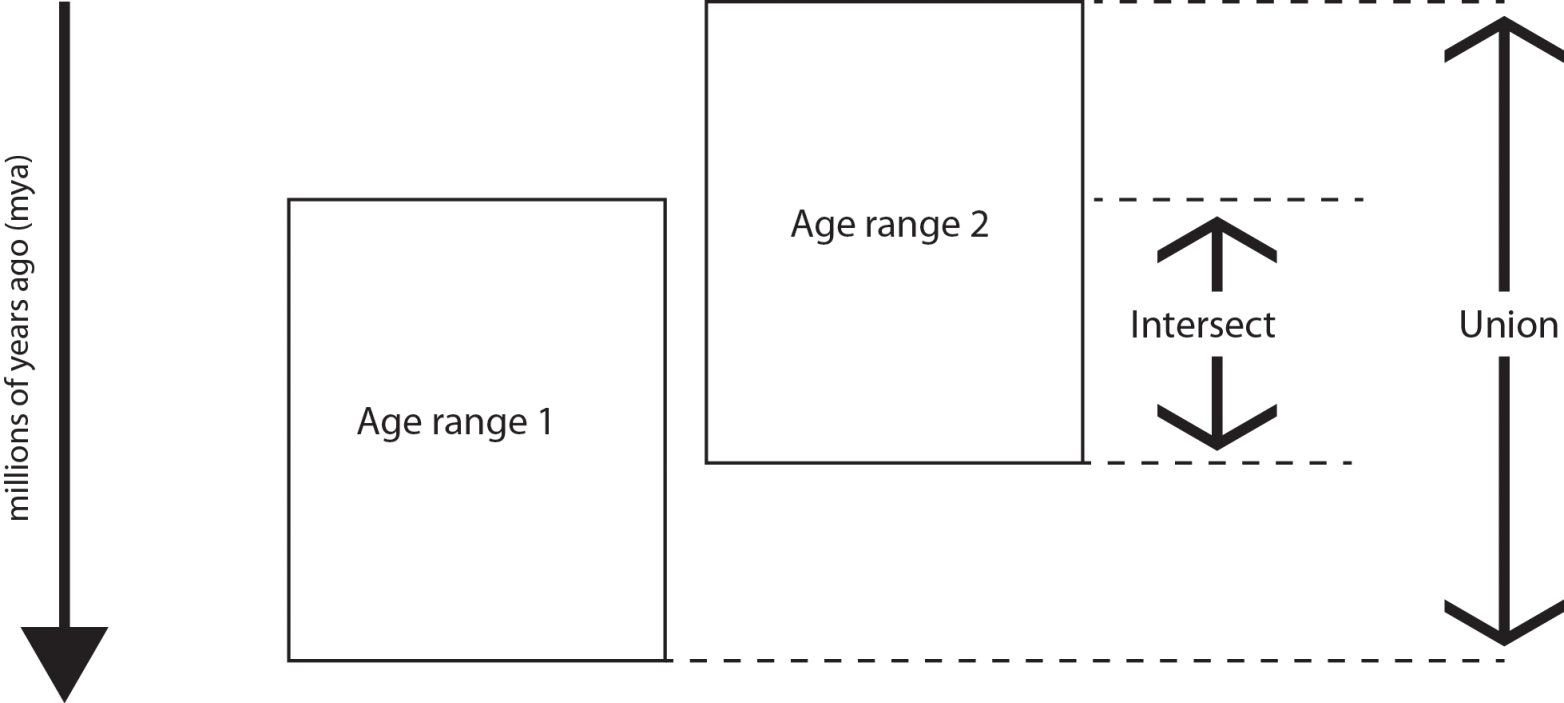
Calculation of the union and intersect.

**Figure 6.**
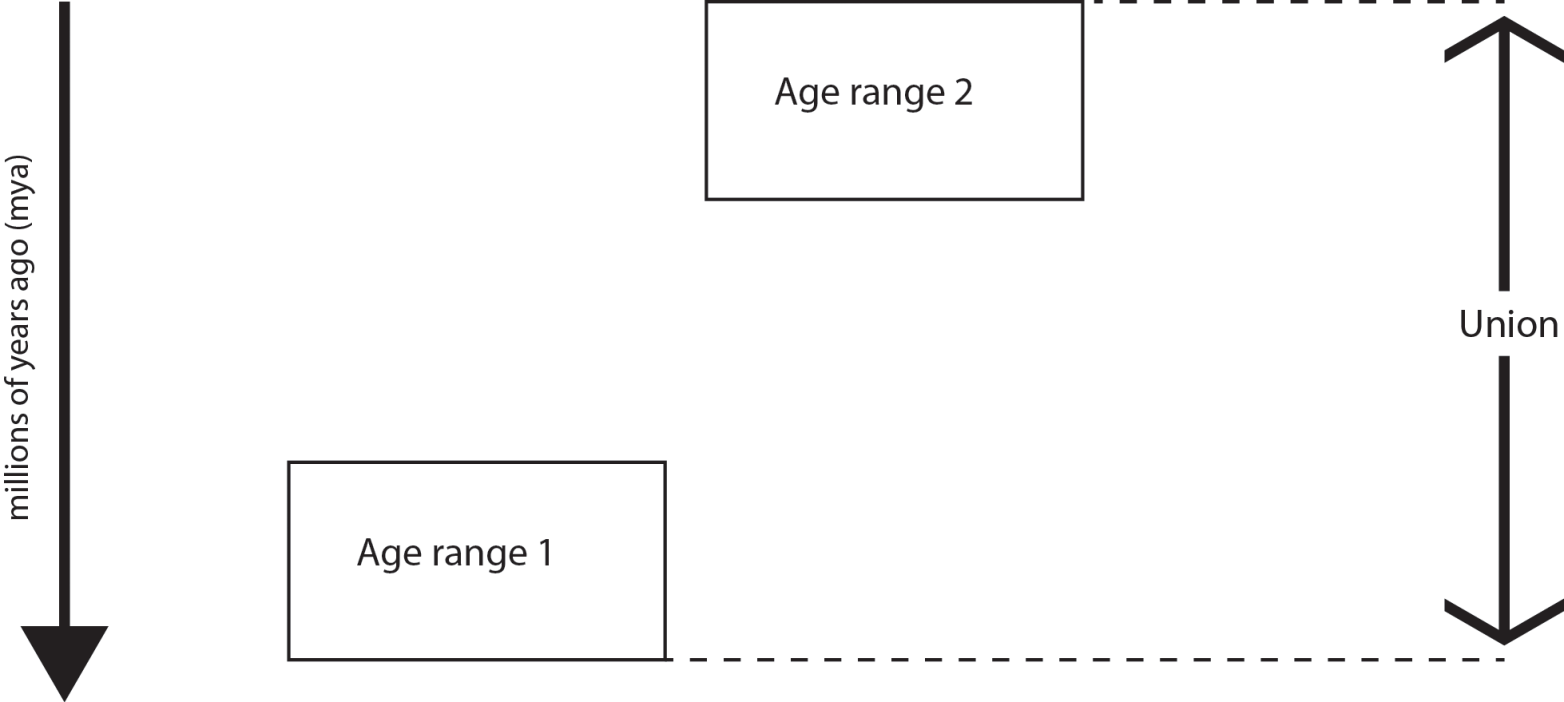
When there is no overlapping time periods the intersect is undefined.

The union gives the maximum possible time range for the species and be calculated for all GeoTime data sets. The intersect gives the overlapping range of the data sets and can only be calculated when there is a time period that is present across the data sets (Figures 5 and 6).

## Scratchpads 2.0 Implementation

The Scratchpads 2.0 implementation of the GeoTime module will allow for a variable number of age ranges (either predefined or custom) with individual references to be recorded. This is an improvement over the Scratchpads 1.0 implementation, which only allowed for one custom age range and a single reference to be given to the geological age datasets as a whole.

## Issues

Given the nature of some geological age data (e.g. chronostratigraphy), it makes sense to associate these nodes with a Drupal taxonomy. In this model the Jurassic period has only one parent, the Mesozoic era, and several children, the Upper, Middle and Lower Jurassic. Attaching age metadata (e.g. top and base ages) to the taxonomy terms allows all records of a given term to be updated with a single change. The current Scratchpads implementation has a mechanism for achieving this but requires a separate content type for extending each taxonomy, plus a separate content type for ages not associated with a taxonomy. It was decided not to use this option due to the proliferation of content types required for sites dealing with multiple types of age data.

## Future plans

We will create functionality to allow content to be searched using geological age data, either by union or intersect. Some example questions that could potentially be answered by this functionality are:

1. Which taxa were alive in age X?

2. Are there specimens of taxon X in age range Y?

3. Which taxa co-existed in time with taxon X?

For both questions 1 and 2 an important part of the functionality is that the age can be expressed in terms of multiple different systems - absolute age in Ma, chronostratigraphic stage or fossil zone. The query function will perform its search by converting both the recorded data, and the query parameters into absolute ages, and then converting again if necessary to display the required results. This allows any kind of primary data to be queried using the same interface and the results to be displayed in any appropriate format.

Scratchpads 2.0 will allow for data to be imported from the GBIF Vocabularies site dynamically, allowing for changes made to the metadata (e.g. base age, top age) of a geological age to be automatically propagated across the Scratchpads, making use of the GeoTime functionality.

Once a system has been created for recording geological age data the next obvious step is to create a way for these data to be displayed visually. One project that has been used to develop a relevant working example of age data is the SIMILE Timeline project (http://www.simile-widgets.org/timeline/); see http://simile.mit.edu/timeline/examples/dinosaurs/dinosaurs2.html for a geological example.

The Timeline widget has already been integrated with the Drupal views module (http://drupal.org/project/timeline) but, as yet, there is no Drupal 7 version. Migrating this code to Drupal 7 and adding support for geological age ranges (as in the above example) would allow for an aesthetically pleasing and easy-to-use visual layer to be applied to the data.

## Going further

One possible use for the functionality developed here is to create a first and last occurrence database for a large number of taxa. This would become a useful resource for calibrating phylogenies (Marshall 2008) and studying changes in biotas through time (for example, Johnson et al. 2008). Additionally, range data can be used in conjunction with phylogenetic studies to help correct for incomplete sampling of the fossil record. This has been shown to alter the apparent nature of diversification in odonates (dragonflies, damselflies and extinct relatives) from an expansionist to a logistic model, with wider implications for palaeodiversity studies (Davis et al. 2011). An online repository of up-to-date range data would facilitate this type of work in future.

Although the functionality described is currently used for recording geological age data, the same functionality could be used to record and display data about other properties that can be measured in ranges, e.g. depth in sediment cores from lakes (e.g. Dalton et al. 2005).

The developed functionality could also be used in archaeological contexts by using new or modified vocabularies.

Moving beyond chronostratigraphy, it would be useful to develop processes to connect lithostratigraphic information into the scratchpad environment taking advantage of the stratigraphic lexicons published by national geological surveys (http://ngmdb.usgs.gov/Geolex) For example the formations found around Lyme Regis (e.g. Black Ven Marl, Belemnite Shales etc.). These could potentially be entered as synonyms of existing named time intervals, or added as a separate vocabulary. This method would allow for local stratigraphic data to be recorded in the Scratchpad system. An extended dataset of this nature would make it easier to integrate the Scratchpads with existing local, regional and global databases.
